# Psychosocial functioning in adolescents growing up with chronic disease: The Dutch HBSC study

**DOI:** 10.1007/s00431-021-04268-9

**Published:** 2021-09-30

**Authors:** Emma E. Berkelbach van der Sprenkel, Sanne L. Nijhof, Geertje W. Dalmeijer, N. Charlotte Onland-Moret, Simone A. de Roos, Heidi M. B. Lesscher, Elise M. van de Putte, Cornelis K. van der Ent, Catrin Finkenauer, Gonneke W. J. M. Stevens

**Affiliations:** 1 Department of Pediatrics, Wilhelmina Children’s Hospital, University Medical Center, Utrecht University, Utrecht, The Netherlands; 2 Julius Centre for Health Sciences and Primary Care, University Medical Center Utrecht, Utrecht University, Utrecht, The Netherlands; 3 The Netherlands Institute for Social Research (SCP), Ministry of Health, Welfare and Sport, The Hague, The Netherlands; 4 Department of Animals in Science and Society, Division of Behavioural Neuroscience, Faculty of Veterinary Medicine, Utrecht University, Utrecht, The Netherlands; 5 Department of Interdisciplinary Social Science, Faculty of Social and Behavioural Sciences, Utrecht University, Utrecht, The Netherlands; 6 Department of Pediatric Pulmonology, Wilhelmina Children’s Hospital, University Medical Center Utrecht, Utrecht University, Utrecht, The Netherlands

**Keywords:** Adolescents, Chronic disease, Psychosocial, Functioning

## Abstract

Many adolescents worldwide (indirectly) grow up with a chronic disease, which may impact their functioning and wellbeing. The objective of this study is to assess whether adolescents with a (family member with a) chronic disease differ from their healthy counterparts in terms of psychosocial functioning. Data from the Dutch 2013 HBSC-survey were used, including 7168 adolescents (*Mean*_age_ = 13.7, SD = 1.57, 50.5% female). Participants indicated whether they or one of their family members had a long-term (> 3 months) disease or disability (mental/physical) and were categorized into four groups based on disease presence (none, other, self, both). Psychosocial functioning was assessed in terms of life satisfaction, self-rated health, psychosomatic health, mental health problems, support, substance use, physical exercise, screen time, and school liking. Chronically diseased adolescents (*n* = 162) reported lower life satisfaction, self-rated and psychosomatic health, more mental health problems, lower peer support, more substance use, and less physical exercise compared to healthy peers. Chronically diseased adolescents who also had a family member with a chronic disease (*n* = 74) showed comparable outcomes on these life domains, although they did not differ from their healthy peers regarding peer support, substance use, and physical activity. Healthy adolescents with a chronically diseased family member (*n* = 737) reported significantly lower life satisfaction, self-rated and psychosomatic health, more mental health problems, and less family support compared to healthy peers who grew up in healthy families; however, they reported more positive outcomes than adolescents who had a chronic disease themselves.

*Conclusion*: Having a (family member with a) chronic disease is associated with impaired psychosocial functioning on various life domains. Our findings aid in understanding the psychosocial associates of chronic disease and imply that caregivers should be observant of psychosocial problems among vulnerable adolescents to provide appropriate guidance.

**Table:** 

**What is Known:** *• Adolescents who grow up with a (family member with a) chronic disease encounter numerous challenges that may be related to poorer developmental outcomes on the long term.*	
**What is New:** *• This study adds a comprehensive overview of the psychosocial functioning of adolescents with a (family member with a) chronic disease, as compared to healthy counterparts that grow up in a healthy family.*

**What is Known:**

*• Adolescents who grow up with a (family member with a) chronic disease encounter numerous challenges that may be related to poorer developmental outcomes on the long term.*

**What is New:**

*• This study adds a comprehensive overview of the psychosocial functioning of adolescents with a (family member with a) chronic disease, as compared to healthy counterparts that grow up in a healthy family.*

## Introduction

The progress made in the treatment of pediatric chronic diseases is revolutionary [1]. Many chronic childhood diseases, such as auto-immune disorders, congenital heart disease, and childhood cancer, can now be stabilized or even cured [1–4]. As a result, the percentage of children growing up with a chronic disease has increased considerably, with increases from 12.8% in 1994 to 26.2% in 2006 in the USA [1, 5]. Recent Dutch research has shown that 26% of the children and young adults under the age of twenty-five suffer from a chronic disease, which adds up to a total of more than 1.3 million in the Netherlands [6]. This poses novel challenges for healthcare providers in delivering care for chronically ill children, adolescents, and their families.

Growing up with a chronic disease may impact the psychosocial functioning of the individual and family functioning beyond the actual illness itself [4, 7–10]. Particularly during adolescence, the period of physical and emotional maturation, socialization, and identity formation, management of a chronic disease may constitute a major challenge [11]. Adolescents with a chronic disease experience physical disabilities, periods of hospitalization, disease-related symptoms (such as pain or fatigue), stressful situations, and emotional distress [9, 12]. Related to this, mental health problems, impaired social functioning, and stigmatization have been observed in adolescents with chronic physical conditions [9, 12–15]. Besides that, elevated levels of internalizing and externalizing problems have been found within this population [16]. Retrospective research has indicated that young adults with pediatric chronic illness are less successful in achieving developmental milestones compared to healthy peers [12]. Thus, research suggests that chronic disease affects adolescent functioning in a variety of life domains, yet a comprehensive picture of which life domains are affected and to what extent is lacking.

Rather than being limited to the individual patient, chronic disease of a family member may have spillover effects to other family members. It may impact family functioning in terms of physical, emotional, social, and financial stress and may affect daily activities—like caregiving—and family dynamics [17–20]. For example, having a chronically ill parent is related with increased stress, internalizing problems, substance use, and risk of poorer developmental outcomes for adolescents [21–26]. Similarly, siblings of chronically ill children have been found to be at increased risk for psychosocial problems and impaired cognitive and academic development [27–29]. Although spillover effects of chronic disease have been recognized, to our knowledge, there is a lack of studies that compare psychosocial functioning of healthy adolescents with that of adolescents with a chronic disease themselves and/or with a family member with a chronic disease.

Notwithstanding the previous, there are large inter-individual differences in adolescents’ resilience, and not all adolescents with a chronic disease experience negative consequences. It is therefore of paramount importance to better understand the interplay between chronic illness and psychosocial functioning among adolescents [8]. The objective of this study is to assess whether and how adolescents with a (family member with a) chronic disease differ from their healthy counterparts. Specifically, we distinguished four groups of adolescents: adolescents from healthy families (in the following labelled as *none*), adolescents with a chronically diseased parent or sibling (*other*), adolescents suffering from a chronic disease themselves (*self*), and chronically diseased adolescents with a chronically diseased parent or sibling (*both*). By comparing healthy and diseased adolescents in families with or without family members that suffer from a chronic disease, we can discriminate between associations that are specific to one’s own or family disease. Based on the available empirical research, we hypothesized that compared to no disease, both having a chronic disease and having a family member with a chronic disease is associated with increased psychosocial distress on virtually all life domains. Insights in adolescents’ own perception of their social context and functioning across different life domains are indispensable for effective preventative or therapeutic strategies to improve coping and resilience.

## Methods

### Participants

Data were used from the 2013 Dutch Health Behavior in School-aged Children (HBSC) survey, which is a large, representative survey on the health, wellbeing, and social context of Dutch adolescents in the age of 11–16 years [30]. A randomized sample was drawn from a list of all regular primary and secondary schools in the Netherlands, which was stratified by urbanity level to ensure population representativeness [30]. At the time data were collected, no ethical approval was deemed necessary as data-collection was perceived as non-intrusive for adolescents. As times have changed, we gained ethical approval from the Ethics Assessment Committee of the Faculty of Social Sciences at Utrecht University (FETC17-079 in 2017) for the Dutch HBSC study. Informed consent was obtained from schools, participants, and their parents, and participation was voluntary. Anonymity and confidentiality were guaranteed to all participants, and data-collection took place under exam conditions [30]. In total, 7168 adolescents from 78 primary schools and 67 secondary schools participated in the study; participants had a mean age of 13.7 ± 1.6 years, 50.5% was a girl, and the majority was of Dutch origin (78%) (for more information about the sample, see Table 1).

**Table 1:** Sociodemographic and individual characteristics of the study population

	**Total (*n* = 7168)**	**None (*n* = 6195)**	**Other (*n* = 737)**	**Self (*n* = 162)**	**Both (*n* = 74)**	
**Age, *M* (SD)**	13.68 (1.57)	13.66 (1.56)	13.77 (1.56)	13.75 (1.65)	13.60 (1.60)	*F* = 1.191, *p* = .311
**Sex, *n* (%)**						χ^2^ = 11.829, *p* = .008
Male	3550 (49.5%)	3094 (49.9%)	330 (44.8%)	93 (57.4%)	33 (44.6%)	
Female	3618 (50.5%)	3101 (50.1%)	407 (55.2%)	69 (42.6%)	41 (55.4%)	
**Ethnicity, *n* (%)**						χ^2^ = 20.368, *p* = .498
Dutch	5600 (78.2%)	4835 (78.1%)	569 (77.2%)	138 (85.2%)	58 (78.4%)	
Surinamese	169 (2.4%)	140 (2.3%)	21 (2.8%)	5 (3.1%)	3 (4.1%)	
Antillean	96 (1.3%)	82 (1.3%)	12 (1.6%)	2 (1.2%)	0 (0%)	
Moroccan	192 (2.7%)	164 (2.6%)	25 (3.4%)	1 (0.6%)	2 (2.7%)	
Turkish	238 (3.3%)	210 (3.4%)	23 (3.1%)	2 (1.2%)	3 (4.1%)	
Other Non-Western	458 (6.4%)	406 (6.6%)	45 (6.1%)	6 (3.7%)	1 (1.4%)	
Other Western	412 (5.7%)	355 (5.7%)	42 (5.7%)	8 (4.9%)	7 (9.5%)	
**Living situation, *n* (%)**						χ^2^ = 9.967, *p* = .019
With both parents	5348 (74.6%)	4654 (75.1%)	518 (70.3%)	125 (77.2%)	51 (68.9%)	
**SES, *n* (%)**						χ^2^ = 30.088, *p* < .001
Low	922 (12.9%)	780 (12.6%)	106 (14.4%)	21 (13.0%)	15 (20.3%)	
Middle	3957 (55.2%)	3370 (54.4%)	456 (61.9%)	92 (56.8%)	39 (52.7%)	
High	2289 (31.9%)	2045 (33.0%)	175 (23.7%)	49 (30.2%)	20 (27%)	
**School level, *n* (%)**						χ^2^ = 4.087, *p* = .906
Primary school	1597 (22.3%)	1391 (22.5%)	147 (19.9%)	39 (24.1%)	20 (27%)	
Vocational education	1372 (19.1%)	1176 (19%)	146 (19.8%)	33 (20.4%)	17 (23%)	
Intermediate secondary education	1511 (21.1%)	1297 (20.9%)	165 (22.4%)	36 (22.2%)	13 (17.6%)	
Higher secondary education	1465 (20.4%)	1267 (20.5%)	152 (20.6%)	30 (18.5%)	16 (21.6%)	
Pre-university education	1223 (17.1%)	1064 (17.2%)	127 (17.2%)	24 (14.8%)	8 (10.8%)	

### Measures

#### Chronic disease of the adolescent and in the family

Participants indicated whether they or one of their family members (sibling, parent) had a long-term (> 3 months) disease or disability (mental or physical). Based on their responses, all participants were categorized into four groups based on the presence of chronic disease within their family: none (*n* = 6195), other (*n* = 737), self (*n* = 162), both (*n* = 74).

#### Demographic information and individual characteristics

Self-reported data was collected for age, sex, ethnicity (based on the country of birth of the parents), living situation (with both parents or not), and participants’ school level (primary school, vocational education, intermediate secondary education, higher secondary education, pre-university education). Family socio-economic status (SES) was assessed with the Family Affluence Scale (FAS), assessing family material prosperity [30, 31]. International studies have shown that the FAS is a valid and reliable measure of SES [31].

#### Psychosocial functioning in different life domains

To assess adolescent psychosocial functioning in a comprehensive manner, nine life domains were identified using various items and validated scales: life satisfaction, self-rated health, psychosomatic health, mental health, support, substance use, physical exercise, screen time, and school liking (for details about the wording, values and Cronbach’s alpha, see Table 2).

**Table 2:** Psychosocial life domains, corresponding HBSC items/subscales and internal consistency measurements

**Domain**	**Incorporated items/subscales**	**Values**	**Cronbach’s alpha**
**Life satisfaction**	How do you feel about your life?	0 (worst possible life) to 10 (best possible life)	N/A
**Self-rated health**	What do you think of your own health?	Bad; reasonable; good; excellent	N/A
**Psychosomatic health**	In the last 6 months, how often have you had/felt: - Headache - Stomach ache - Back ache - Dizzy - Difficulties getting to sleep - Unhappy - Moody - Nervous	About every day; more than once a week; about every week; about every month; rarely or never	.798
**Mental health problems**	SDQ subscale conduct problems (5 items)	Range 0–10: higher score = more problems	.475
	SDQ subscale hyperactivity-inattention (5 items)	Range 0–10: higher score = more problems	.731
	SDQ subscale emotional problems (5 items)	Range 0–10: higher score = more problems	.702
	SDQ subscale peer problems (5 items)	Range 0–10: higher score = more problems	.470
**Support**	HBSC subscale support at home (4 items)	Range 1–7: higher score = more support at home	.901
	HBSC subscale support from friends (4 items)	Range 1–7: higher score = more support from friends	.927
**Substance use**	- Lifetime prevalence smoking - Lifetime prevalence cannabis - Monthly prevalence alcohol (in the last 30 days)	Yes or no	.710
**Physical exercise**	Over the past 7 days, on how many days were you physically active for a total of at least 60 min per day?	0–7 days	N/A
**Screen time**	- About how many hours a day do you usually watch television in your free time? - About how many hours a day do you usually play games on a computer or game console in your free time?	None at all; about half an hour a day; about 1 h a day; about 2 h a day; about 3 h a day; about 4 h a day; about 5 h a day; about 7 or more hours a day	.809
**School liking**	How do you feel about school at present?	I don’t like it at all; I don’t like it very much; I like it a bit; I like it a lot	N/A

Several of these instruments were validated in previous research. The Strengths and Difficulties Questionnaire (SDQ) distinguishes between hyperactivity-inattention, emotional symptoms, peer problems, and conduct problems. Previous research in Dutch samples showed measurement invariance of the SDQ subscales for different groups (i.e., boys/girls, immigrants/non-immigrants, adolescents with different educational levels) and acceptable internal consistency, test–retest stability, and concurrent validity [32, 33]. An 8-item scale was used to assess psychosomatic health complaints. This instrument has good psychometric properties and has been validated as an unbiased measurement of subjective psychosomatic health complaints across age groups, sex, SES, and countries [34]. Moreover, the scales on experienced support from family and friends showed good internal reliability, good test–retest reliability, and moderate construct validity in previous studies—also among adolescents and populations with chronic disease [35, 36].

### Statistical analysis

All analyses were performed using SPSS for Macintosh version 24.0 (IBM, Armonk, NY). To assess (demographic) differences between the different groups established based on the presence of a chronic disease, χ^2^ tests were performed for categorical data (sex, ethnicity, living situation, SES, and school level) and one-way analysis of variance (ANOVA) for continuous data (age). The variables for which differences were found were included as control variables in the subsequent analyses. Next, to examine differences between the four groups regarding their psychosocial functioning in the different life domains, univariate analyses of variance were performed. To facilitate the interpretation of significant outcomes between the groups, estimated marginal means were used to obtain pairwise comparisons of all groups. Bonferroni adjusted probabilities were used for all pairwise comparisons. Subsequently, mean differences, standard errors for the differences, and 95% confidence interval for all comparisons were assessed. Z-scores were calculated to enable (graphic) comparison of the different domains by standardizing the distributions.

## Results

**Fig. 1:**
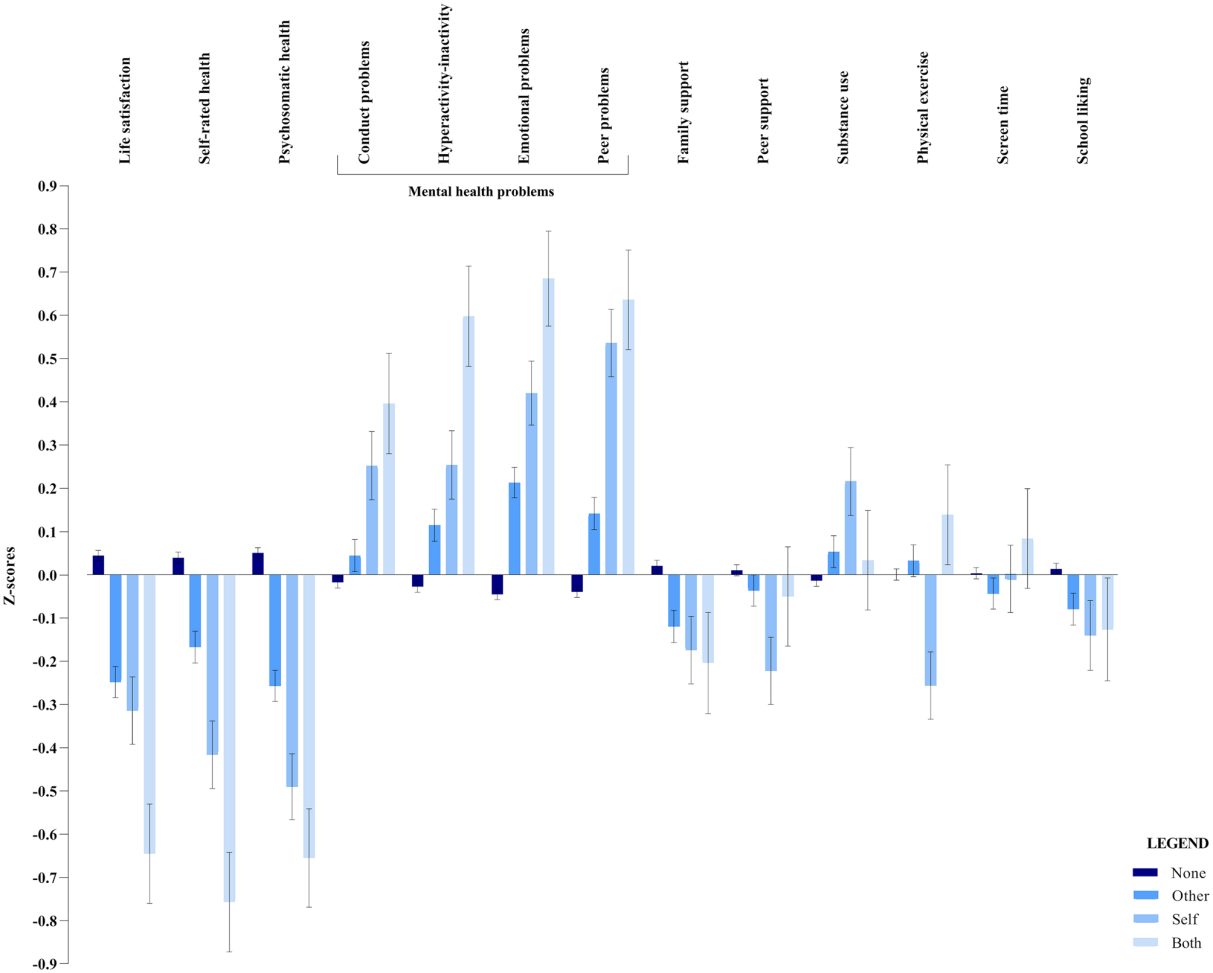
Average z-scores and error bars showing the self-reported psychosocial functioning per life domain among adolescents growing up with chronic disease

Table 1 presents the sociodemographic and individual characteristics for the four groups. All four groups were comparable with respect to age, ethnicity and school level. We observed a significant difference in terms of sex (*χ*^2^ = 11.829, *p* = 0.008), living situation (*χ*^*2*^ = 9.967, *p* = 0.019), and SES (*χ*^2^ = 30.088, *p* < 0.001). Therefore, sex, living situation, and SES were added as potential confounders in all analyses. The results from the analyses of variance assessing differences in psychosocial functioning between four groups of chronic disease are reported in Table 3 and are visualized with z-scores and error bars in Fig. 1. The effect sizes (η^2^ ~ 0.01) are consistent across several domains at the cost of adolescents who (indirectly) grow up with chronic disease, which underlines the clinical relevance of our analyses.

**Table 3:** Univariate analyses of variance assessing differences in psychosocial functioning between four groups of chronic disease

	**None (*n* = 6195)**			**Other (*n* = 737)**			**Self (*n* = 162)**			**Both (*n* = 74)**			**Univariate tests**
	**M**	**SE**	**95% CI**	**M**	**SE**	**95% CI**	**M**	**SE**	**95% CI**	**M**	**SE**	**95% CI**	
**Life satisfaction**	7.78^a^	.02	7.74–7.82	7.31^b^	.06	7.19–7.42	7.20^bc^	.13	6.95–7.45	6.67^c^	.19	6.30–7.03	*F* (3,6930) = 35.918, *p* < .001, η^2^ = .015
**Self-rated health**	3.24^a^	.01	3.22–3.25	3.09^b^	.03	3.04–3.14	2.92^c^	.06	2.81–3.02	2.68^c^	.08	2.52–2.84	*F* (3,6968) = 33.970, *p* < .001, η^2^ = .014
**Psychosomatic health**	4.09^a^	.01	4.07–4.11	3.85^b^	.03	3.80–3.91	3.67^c^	.06	3.55–3.78	3.54^c^	.09	3.36–3.71	*F* (3,7085) = 47.863, *p* < .001, η^2^ = .020
**Conduct problems**	1.82^a^	.02	1.74–1.86	1.91^ab^	.06	1.80–2.02	2.23^bc^	.12	2.00–2.46	2.45^c^	.18	2.10–2.79	*F* (3,7035) = 8.357, *p* < .001, η^2^ = .004
**Hyperactivity-inattention**	3.94^a^	.03	3.88–4.00	4.28^b^	.09	4.12–4.46	4.62^bc^	.19	4.24–5.00	5.45^c^	.28	4.90–6.01	*F* (3,7030) = 16.955, *p* < .001, η^2^ = .007
**Emotional problems**	2.44^a^	.03	2.39–2.49	3.02^b^	.08	2.87–3.18	3.49^bc^	.17	3.16–3.82	4.09^c^	.25	3.61–4.58	*F* (3,7035) = 40.774, *p* < .001, η^2^ = .017
**Peer problems**	1.55^a^	.02	1.51–1.59	1.84^b^	.06	1.73–1.96	2.47^c^	.13	2.23–2.72	2.63^c^	.18	2.27–2.99	*F* (3,7033) = 33.869, *p* < .001, η^2^ = .014
**Family support**	5.96^a^	.02	5.93–5.99	5.77^b^	.05	5.68–5.87	5.70^ab^	.10	5.50–5.90	5.66^ab^	.15	5.36–5.96	*F* (3,7124) = 7.091, *p* < .001, η^2^ = .003
**Peer support**	5.76^a^	.02	5.72–5.79	5.69^ab^	.05	5.60–5.79	5.44^b^	.12	5.23–5.65	5.67^ab^	.16	5.37–5.98	*F* (3,6989) = 3.321, *p* < .001, η^2^ < .001
**Substance use**	.15^a^	.004	.14–.16	.17^ab^	.01	.15–.19	.22^b^	.02	.17–.26	.16^ab^	.03	.10–.23	*F* (3,7144) = 3.679, *p* = .012, η^2^ = .002
**Physical exercise**	4.33^a^	.03	4.28–4.38	4.39^a^	.07	4.25–4.54	3.83^b^	.15	3.52–4.13	4.60^a^	.23	4.16–5.05	*F* (3,7066) = 4.307, *p* = .005, η^2^ = .002
**Screen time**	3.20	.02	3.16–3.24	3.11	.06	2.30–3.24	3.18	.14	2.91–3.44	3.34	.20	2.94–3.73	*F* (3,7123) = .693, *p* = .556, η^2^ = .000
**School liking**	3.17	.01	3.15–3.19	3.10	.03	3.04–3.16	3.05	.06	2.87–3.25	3.06	.09	2.87–3.25	*F* (3,6865) = 3.279, *p* = .020, η^2^ = .001

^*^Labels: none = adolescents from healthy families, other = adolescents with a chronically diseased parent or sibling, self = adolescents suffering from a chronic disease themselves, and both = chronically diseased adolescents with a chronically diseased parent or sibling. Different letters (superscript a/b/c) behind the means of two groups indicate significant differences between groups

### Life satisfaction and self-rated health

All adolescents who grew up with chronic disease (*other*, *self*, *both*) reported significantly poorer outcomes in terms of life satisfaction and self-rated health compared to their healthy peers (*none*). Nevertheless, adolescents who had a chronic disease themselves and had a family member with a chronic disease (*both*) reported significantly lower life satisfaction compared to adolescents who had a chronically diseased family member (*other*). For self-rated health, our findings showed that the two groups of adolescents who had a chronic disease themselves (*self*, *both*) view their health significantly worse than those who grew up with a chronically diseased family member (*other*).

### Psychosomatic health

Psychosomatic health complaints were reported significantly more among all adolescents who grew up with chronic disease (*other*, *self*, *both*) as compared to their healthy peers (*none*). Moreover, our findings indicate that adolescents who had a chronic disease themselves (*self*, *both*) reported even more psychosomatic health complaints than healthy adolescents who grew up with a chronically diseased family member (*other*).

### Mental health

Generally, adolescents who grew up with chronic disease (*other*, *self*, *both*) reported significantly more conduct problems, hyperactivity-inactivity, emotional problems, and peer problems than their healthy peers (*none*). The only exception was conduct problems, for which no differences between healthy adolescents (*none*) and those with a family member with chronic disease (*other*) were found. Adolescents who had a chronic disease themselves and had a family member with a chronic disease (*both*) reported more problems on all four indicators of mental health than adolescents in the *other* group. Adolescents who had a chronic disease themselves (*self*) reported similarly as adolescents in the *both* group; only for peer problems the latter (*both*) reported more problems than the former (*self*) group.

### Support

For support, results showed a different pattern than for the abovementioned (mental) health indicators. In terms of family support, adolescents with a family member with a chronic disease (*other*) showed significantly poorer outcomes compared to their healthy peers (*none*), while no differences were found between the other groups of adolescents. When assessing peer support, adolescents with a chronic disease themselves (*self*) reported significantly worse outcomes than their healthy peers (*none*). Again, no differences between the other groups were revealed.

### Lifestyle factors and school liking

Adolescents who grew up with a chronic disease (*self*) scored significantly poorer on physical exercise compared to all other groups (*none*, *other*, *both*), as well as in terms of substance use (alcohol, smoking, cannabis) compared to their healthy peers (*none*). With regards to screen time and school liking, we did not find significant differences between any of the groups.

## Discussion

The purpose of this study was to assess the psychosocial functioning of adolescents who grow up with (a family member with) a chronic disease. Psychosocial functioning across a range of life domains were compared between adolescents who were (directly and/or indirectly) exposed to chronic disease and healthy adolescents in healthy families. Our results showed that adolescents with a chronic disease themselves (*self*) had decrements in many life domains. More specifically, and in line with former research, they reported lower life satisfaction, self-rated health and psychosomatic health, more conduct problems, hyperactivity-inactivity, emotional problems and peer problems, lower peer support, more substance use, and less physical exercise when compared to their healthy peers. Chronically diseased adolescents who also had a chronically diseased family member (*both*) showed similar scores across all life domains as adolescents with a chronic disease themselves (*self*), although it must be noted that no differences in peer support, substance use, and physical exercise between the former group and the group of healthy peers were found. These findings suggest that there is no additional risk for impaired psychosocial functioning when an adolescent with a chronic disease also has a chronically diseased family member (sibling or parent). However, this does not mean that having a chronically diseased family member is not associated with less optimal outcomes. Although healthy adolescents with a chronically diseased family member (*other*) often reported more positive outcomes than the groups of adolescents with a chronic disease (*self, both*), significantly lower life satisfaction, self-rated and psychosomatic health, more hyperactivity-inactivity, emotional problems and peer problems, and less family support were found in this group as compared to healthy peers in healthy families (*none*).

Our results show that growing up with a chronic disease in adolescence is associated with impaired psychosocial functioning. The decremental psychosocial associates of chronic disease may be related to a great range of factors that have been studied previously. Foremost, having a chronic disease exposes the adolescent to numerous physical, emotional, psychological, and social challenges [9, 12, 37]. Due to their disease, adolescents may be unable to fully participate in school, leisure activities, or social events, which may go hand in hand with an increased incidence of psychological adjustment problems, lower self-esteem, stress, and stigmatization [9, 38, 39]. These challenges may be particularly prominent during adolescence, as one of the most important developmental demands in adolescence is to become independent of one’s parents and to develop peer relationships. The aforementioned problems within various life domains may impede normal functioning and development but may also persist beyond adolescence, thereby posing a risk for physical, emotional and social well-being across the lifespan [10]. Hereby, the need to prevent chronic disease where possible and promote healthy behavior among adolescents is emphasized. Furthermore, caregivers should—next to the physical aspects of a disease—be aware and observant of the psychosocial problems that their patients may encounter, and development of preventative interventions that nourish psychosocial wellbeing and resilience may be of great value [10, 40].

In addition to having a chronic disease oneself, having a family member (sibling or parent) with a chronic disease was associated with less optimal psychosocial functioning, even if the adolescents did not suffer from a disease themselves. This apparent spillover effect has been previously linked to the emotional impact, increased amount of physical, social and financial stress, lack of attention or guidance (caregiving), altered roles within the family, and limited resources [17, 21, 26, 29]. In line with the finding that these adolescents reported lower family support than their healthy peers, these adolescents may take on a caregiver role early on in life, which may be accompanied with emotional challenges, withhold them from engaging in normal daily-life activities, and complicate interactions with peers [26, 41]. In future studies, it would be interesting to further investigate the differences between having a mother, father, or sibling with a chronic disease. Remarkably, girls more often report having a chronically diseased family member, which may be related with gender-specific differences, such as individual characteristics (sensitivity to signals of need for care), family roles, or tendency to report others’ chronic disease [41, 42]. In order to safeguard normal development of adolescents and provide adequate support when necessary, caregivers should be attentive to the potential impact of growing up with chronically diseased family member [41].

Even though adolescents who were confronted with chronic disease on average scored worse on most life domains compared to their healthy peers, we also observed considerable variance, especially in the group where both the adolescent and a family member suffered from a chronic disease (*both*). It is vital to further study which adolescents are specifically at higher risk to lag behind in comparison to their peers or experience increased distress. Differences across and within patient populations may help us to understand what determines an individual’s risk or resilience to the psychosocial impact of chronic disease.

To our knowledge, this is the first study to evaluate the self-reported psychosocial functioning of adolescents with a (family member with a) chronic disease by comparing psychosocial functioning of healthy adolescents with that of adolescents with a chronic disease themselves and/or within the family. The sample is representative for the Dutch population and provides insight into various life domains by means of frequently used and validated measures. However, this study also has several limitations. Given its cross-sectional design, causal conclusions cannot be drawn. Especially since chronic diseases may also primarily affect mental health, impaired self-reported psychosocial functioning may be a consequence and/or a predicting factor of chronic disease. Future longitudinal research is warranted to shed more light into this potentially bidirectional association. Also, the risks that adolescents with a (family member with a) chronic disease show in different life domains may be interrelated, e.g. adolescents may report lower life satisfaction because they reported more mental health problems. Furthermore, no information was obtained about the type of chronic disease adolescents or their family members suffered from. Associations between chronic disease and psychosocial functioning may vary according to the type of chronic disease, as specific diseases vary considerably on aspects that shape and differentially affect adolescent functioning (e.g., life-threatening, physical disability, mental health problems) [10, 28]. Future research should be performed to assess whether the observed patterns differ among specific (patient) populations. Also, the proportion of adolescents who reported to grow up with a chronic disease in our sample was considerably lower (3.3%) than estimated by medical caretakers (26.2%)[1, 6, 37]. From our results, we can conclude that perceiving oneself as chronically ill is associated with impaired psychosocial functioning on various life domains. Thus, one might be able to distinguish a group that is more vulnerable as compared to healthy peers by assessing self-reported presence of chronic illness. Future research should evaluate the association between ‘objective’ chronic disease as assessed by physicians and adolescents’ perception of a chronic disease, and which factors influence discrepancies between both. Moreover, several domains that may affect psychosocial functioning among adolescents (with a chronic disease)—such as school functioning, resilience, and sleep—were not assessed as they were not incorporated in the HBSC questionnaire. It would be interesting to further explore these aspects in future studies. Lastly, the study only included participants who were able to attend school. Given that at least some adolescents with a chronic disease may not be present or attend special education, this may impede the external validity of our study. Follow-up studies should therefore attempt to obtain more information regarding the chronic disease type and duration, and to include patients who may not be able to attend school as well.

## Conclusion

The present study provides evidence that having a (family member with a) chronic disease is associated with impaired psychosocial functioning on various life domains. We found that chronically diseased adolescents (*self*) reported significantly lower life satisfaction, self-rated health and psychosomatic health, more mental health problems, lower peer support, more substance use, and less physical activity when compared to healthy peers. Moreover, chronically diseased adolescents who also had a chronically diseased family member (*both*) showed comparable outcomes on most of these life domains. Lastly, we found evidence of a spillover effect of growing up healthy with a chronically diseased family member (*other*), as these adolescents reported significantly lower life satisfaction, self-rated health and psychosomatic health, more mental health problems, and less family support compared to their healthy peers who grew up in healthy families (*none*). Our findings may aid in understanding the psychosocial associates of chronic disease and imply that—in order to safeguard healthy development—caregivers should be observant of psychosocial problems among adolescents who (indirectly) grow up with chronic disease.

## Data Availability

The data is available for external use by agreement with the HBSC International Coordinator and the Principal Investigators. Information on how to request further data can be found on www.hbsc.org.

## Abbreviations

FAS: Family Affluence Scale
HBSC: Health Behavior in School-aged Children
SDQ: Strengths and Difficulties Questionnaire
SES: Socio-Economic Status
